# Hypermethylation of the SEPT9 Gene Suggests Significantly Poor Prognosis in Cancer Patients: A Systematic Review and Meta-Analysis

**DOI:** 10.3389/fgene.2019.00887

**Published:** 2019-09-19

**Authors:** Na Shen, Ting Wang, Delei Li, Yaowu Zhu, Huaping Xie, Yanjun Lu

**Affiliations:** ^1^Department of Laboratory Medicine, Tongji Hospital, Tongji Medical College, Huazhong University of Science and Technology, Wuhan, China; ^2^Department of Gastroenterology, Tongji Hospital, Tongji Medical College, Huazhong University of Science and Technology, Wuhan, China

**Keywords:** cancer, *Septin 9 (SEPT9)*, methylation, prognosis, biomarker, meta-analysis

## Abstract

**Background:** Aberrant hypermethylation of the *Septin 9 (SEPT9)* is an early event in several human cancers, and increasing studies have reported good performance of methylated *SEPT9* (*mSEPT9*) in cancer diagnosis. Recent studies further focused on its value in cancer prognosis, but results are not clearly elucidated.

**Methods:** A comprehensive search to identify relevant studies about the association between *mSEPT9* and cancer prognosis was conducted through the EMBASE, PubMed, and Web of Science databases (up to January 2019). The main outcomes were overall survival (OS) and disease-free survival (DFS). The hazard ratio (HR) and 95% confidence interval (CI) for OS and DFS were extracted from each included study and pooled using a random-effects model.

**Results:** Ten eligible studies comprising 1,266 cancer patients were included. Results demonstrated that *mSEPT9* was associated with poor OS (HR = 2.07, 95% CI = 1.40–3.06). Specially, *mSEPT9* detected in preoperative plasma predicted worse OS in cancer patients (HR = 3.25, 95% CI = 1.93–5.48). In addition, we also identified a significant association of *mSEPT9* with decreased DFS of cancer (HR = 3.24, 95% CI = 1.81–5.79).

**Conclusion:** Our meta-analysis supports that *mSEPT9* is associated with reduced OS and DFS in cancer patients. Moreover, detection of *mSEPT9* using plasma appears to be a convenient and promising way to predict long-term survival of cancer patients.

## Introduction

Septins are a conserved group of GTP-binding proteins that play a crucial role in cytokinesis, cytoskeleton, and cell cycle control (Hall and Russell, 2004; Russell and Hall, 2011). As a star member of the *Septin* gene family, *Septin 9 (SEPT9)* is located at chromosome 17q25.3 and demonstrates both oncogenic and tumor-suppressive impacts on human cancers (Connolly et al., 2011; Verdier-Pinard et al., 2017). Previous studies have uncovered that methylated *SEPT9* (*mSEPT9*) is associated with tumorigenesis based on transcriptionally silencing due to aberrant hypermethylation of the CpG island within the *SEPT9* promoter (Connolly et al., 2011; Wasserkort et al., 2013; Wang et al., 2018). Detection of *mSEPT9* has been reported in several cancers, including colorectal cancer (CRC), head and neck squamous cell carcinoma (HNSCC), and gastric cancer (GC) (Lee et al., 2013; Schrock et al., 2017; Song et al., 2018).

Nowadays, the diagnostic significance of *mSEPT9* has been elucidated in several cancers, and specially, the *mSEPT9* assay (Epi proColon) becomes the first blood-based test approved by U.S. FDA for CRC screening. Some researches further pay attention to the *mSEPT9*’s prognostic performance on cancer. In 2013, Dietrich et al. detected malignant pleural effusions from 58 cases with various cancers and found that *mSEPT9* indicated a poor survival (Dietrich et al., 2013). Subsequently, the association of *mSEPT9* with cancer prognosis was investigated in CRC (Lee et al., 2013; Tham et al., 2014; Freitas et al., 2018; Song et al., 2018), GC (Lee et al., 2013), HNSCC (Schrock et al., 2017), and so on (Kuo et al., 2014; Angulo et al., 2016; Branchi et al., 2016; Jung et al., 2016).

To date, however, the prognostic value of *mSEPT9* in cancer patients has not yet been methodically elucidated. Herein, we performed a systematic review and meta-analysis to summarize the published data and evaluate the prognostic impact of *mSEPT9* on human cancers.

## Materials and Methods

Our meta-analysis was conducted based on the guidelines of the Preferred Reporting Items for Systematic Reviews and Meta-Analyses (PRISMA) (Moher et al., 2009). The PRISMA 2009 checklist is shown in **Supplementary Table S1**.

### Search Strategy

A comprehensive electronic search was performed *via* the EMBASE, PubMed, and ISI Web of Science databases through January 2019 without any restriction. The search items were combinations of “SEPT9,” “mSEPT9,” “septin 9,” “prognosis” and “survival.” There was no language restriction.

### Criteria of Inclusion and Exclusion

Two independent authors conducted the literature search and study selection. Discrepancies were resolved by discussion. Studies were considered eligible if they met the following criteria: (1) cohort studies for evaluating the prognostic role of *mSEPT9* in cancer patients; and (2) studies reporting hazard ratios (HRs) and 95% confidence intervals (CIs) or providing information to estimate HRs. The exclusion criteria were as follows: (1) reviews, meta-analyses, opinion, abstracts, and cellular or animal experiments; and (2) studies with overlapping data. If studies had overlapping data, we kept the one with the larger sample size.

### Data Extraction

Two independent authors extracted the following items from each included study: first author, publication year, country, patient number, sampling time, follow-up, cancer type and stage, detection method, and prognostic outcomes. Outcome measures included overall survival (OS), disease-free survival (DFS), disease-specific survival (DSS), and progression-free survival (PFS).

### Quality Evaluation

Two authors independently conducted quality evaluation, and discrepancies were resolved by discussion. We used the Newcastle-Ottawa scale (NOS) to assess the quality of each included study, with quality score from 0 to 9 (**Supplemental Table S2**) (Stang, 2010). Quality evaluation was not an exclusion criterion for eligible studies.

### Statistical Analysis

Multivariate-adjusted HRs and 95% CIs were preferentially extracted from each included study, if available. If a study did not report the HR and 95% CI, these measures were extrapolated by the method of Parmar and Tierney (Parmar et al., 1998; Tierney et al., 2007). We used the random-effects model (DerSimonian and Laird) to pool these HRs and 95% CIs and examined the heterogeneity by Cochran’s Q test and *I^2^* statistic (Higgins et al., 2003; Harris et al., 2008). *P* < 0.10 or *I^2^* > 50% indicates considerable heterogeneity (Higgins and Thompson, 2002). We also performed subgroup analyses to further evaluate the *mSEPT9*’s prognostic effects based on sample type, sampling time, and cancer type. To assess the stability of pooled results, we applied one-way sensitivity analysis by excluding one study at a time. In addition, the publication bias was examined by Begg’s and Egger’s tests (Begg and Mazumdar, 1994; Egger et al., 1997). All *P* values were two-sided, and *P* ≤ 0.05 was considered significant, unless otherwise specified. All statistical analyses were carried out by Stata 12.1 software (College Station, TX, USA).

## Results

### Study Characteristics

**Figure 1:**
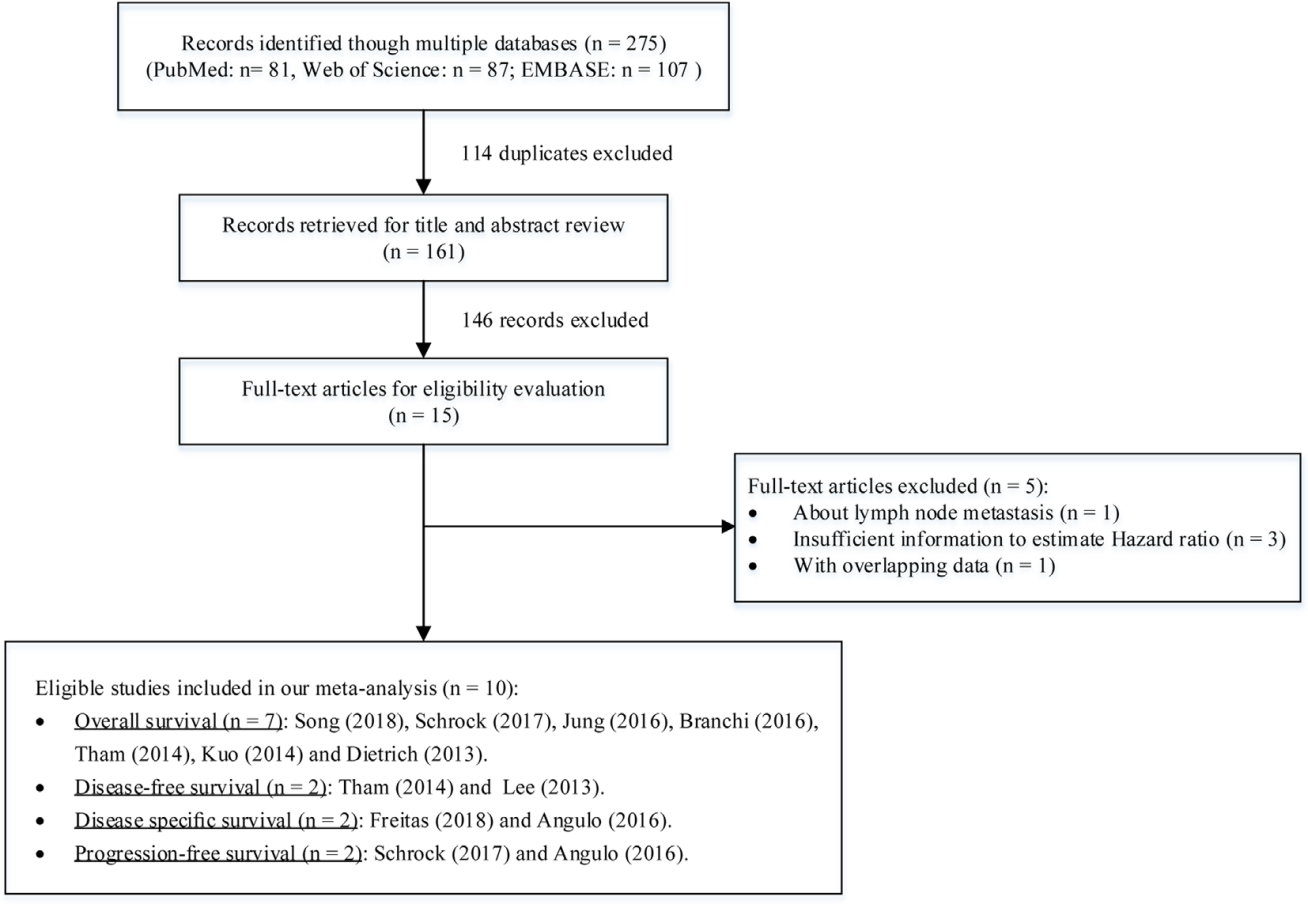
A flowchart of literature search and study selection.

Our search strategy initially obtained 275 records from the PubMed, EMBASE. and Web of Science databases. By title and abstract review, we removed 114 duplicates and 146 records. This large proportion of excluded records consisted of reviews, opinions, conference abstracts, diagnostic studies, *in vitro* studies, and nonhuman studies. Of the remaining 15 full-text publications, five studies were further excluded because of focusing on lymph node metastasis (Nagata et al., 2017), having overlapping data (de Vos et al., 2017), or insufficient information to estimate HRs and 95% CIs (Perez-Carbonell et al., 2014; Villanueva et al., 2015; Chang et al., 2017). Finally, a total of 10 eligible studies were included for this meta-analysis (Dietrich et al., 2013; Lee et al., 2013; Kuo et al., 2014; Tham et al., 2014; Angulo et al., 2016; Branchi et al., 2016; Jung et al., 2016; Schrock et al., 2017; Freitas et al., 2018; Song et al., 2018) (**Figure 1**).

Among these studies including 1,266 cancer patients, seven evaluated the *mSEPT9*’s prognostic significance on OS (Dietrich et al., 2013; Kuo et al., 2014; Tham et al., 2014; Branchi et al., 2016; Jung et al., 2016; Schrock et al., 2017; Song et al., 2018), two evaluated DFS (Lee et al., 2013; Tham et al., 2014), two evaluated DSS (Angulo et al., 2016; Freitas et al., 2018), and two evaluated on PFS (Angulo et al., 2016; Schrock et al., 2017). There were four studies using plasma or serum, of which three collected preoperative samples (Lee et al., 2013; Schrock et al., 2017; Song et al., 2018) and one collected postoperative samples (Tham et al., 2014). Other studies used tissues (Angulo et al., 2016; Branchi et al., 2016; Freitas et al., 2018), ascites (Jung et al., 2016), or pleural effusions (Dietrich et al., 2013). The cancer type comprised CRC (Lee et al., 2013; Tham et al., 2014; Freitas et al., 2018; Song et al., 2018), GC (Lee et al., 2013), HNSCC (Schrock et al., 2017), biliary tract carcinoma (BTC) (Branchi et al., 2016), prostate cancer (PC) (Angulo et al., 2016), esophageal squamous cell carcinoma (ESCC) (Kuo et al., 2014), and multiple cancers (MC) (Dietrich et al., 2013; Jung et al., 2016). The quality of these studies were assessed by NOS score. More details about characteristics of included studies and cancer patients are summarized in **Tables 1** and **2**.

**Table 1:** Characteristics of studies included in this meta-analysis.

Study	Country	Patient number	Sample type	Sampling time	Follow-up	Cancer type	Cancer stage	Detection method	Outcomes	NOS score	HR estimation
Song et al. (2018)	China	99	Plasma	Preoperative	NA	CRC	I–III	Epi proColon 2.0	OS	4	Reported
Freitas et al. (2018)	Portugal	214	Tissues	Postoperative	NA	CRC	I–IV	qPCR	DSS	7	Reported
Schrock et al. (2017) (Training cohort)	Germany	129	Plasma	Preoperative	NA	HNSCC	I–IV	qPCR	OS; PFS	6	Reported
Schrock et al. (2017) (Validation cohort)	Germany	137	Plasma	Preoperative	NA	HNSCC	I–IV	qPCR	OS	6	Reported
Jung et al. (2016)	Germany	81	Ascites	NA	Mean (Range): 141 d (0–832 d); Median (Range): 56 d (0–832 d)	MC	I–IV	qPCR	OS	5	Extrapolated
Branchi et al. (2016)	Germany	71	Tissues	Postoperative	Mean (Range): 23 m (0–104m); Median (Range): 15 m (0–104m)	BTC	I–IV	qPCR	OS	5	Reported
Angulo et al. (2016)	Spain	45	Tissues	Postoperative	NA	PC	I–IV	Golden Gate Methylation Cancer Panel I	DSS; PFS	8	Reported
Tham et al. (2014)	Singapore	150	Serum	Postoperative	Median (Range): 59 m (5–79 m)	CRC	I–III	qPCR	OS; DFS	8	Reported
Kuo et al. (2014)	China	61	Tissues	Postoperative	Mean (Range): 19.6m (1.5–68.0 m)	ESCC	I-IV	Pyrosequencing quantitative methylation assay	OS	5	Extrapolated
Lee et al. (2013)	South Korea	138	Plasma	Preoperative	Mean (Range): 413 d (397—460 d)	GC	I-IV	qPCR	DFS	5	Extrapolated
Lee et al. (2013)	South Korea	83	Plasma	Preoperative	Mean (Range): 518 d (492—543 d)	CRC	I-IV	qPCR	DFS	5	Extrapolated
Dietrich et al. (2013)	Germany	58	Pleural effusions	NA	Mean (Range): 62 d (0–250 d)	MC	NA	qPCR	OS	5	Extrapolated

CRC, colorectal cancer; HNSCC, head and neck squamous cell carcinoma; MC, multiple cancers; BTC, biliary tract carcinoma; PC, prostate cancer; ESCC, esophageal squamous cell carcinoma; GC, gastric cancer; qPCR, quantitative real-time polymerase chain reaction; OS, overall survival; DSS, disease-specific survival; PFS, progression-free survival; DFS, disease-free survival; NOS, Newcastle-Ottawa Scale; d, days; m, months; NA, not available; HR, hazard ratio.

**Table 2:** Characteristics of included patients based on cancer type.

Cancer type	Number of included patients	Number of included studies	Age (years)	Male, n (%)	Stage	*mSEPT9*-positive	Sample type	Sampling time
CRC	546	4	Song et al. (2018): < 40: n = 4; 40–49: n = 11; 50–59: n = 17; 60–69: n = 46; ≥70: n = 21 Freitas et al. (2018): mean (range): 60.35 (25–80) Tham et al. (2014): median (range): 67 (33–88) Lee et al. (2013): mean (SD): 63.59 (11.14)	336 (61.5)	^a^TNM I–IV: I/II: n = 250; III: n = 187; IV: n = 125	NA	Plasma, serum, tissues	Preoperative, Postoperative
MC	139	2	NA	NA	TNM I–IV, patient number of each stage was not provided	31/139 (22%)	Ascites, pleural effusions	NA
HNSCC	266	1	NA	NA	I–IV, patient number of each stage was not provided	NA	Plasma	Preoperative
BTC	71	1	median (range): 63 (36–83)	42 (59)	UICC I: n = 4; UICC II: n = 9; UICC III: n = 28; UICC IV: n = 10; Unknown: n = 20.	16/71 (23%)	Tissues	Postoperative
PC	45	1	mean (SD): 68.7 (7.7)	45 (100)	TNM I–IV, patient number of each stage was not provided	NA	Tissues	Postoperative
GC	138	1	NA	NA	TNM I–IV, patient number of each stage was not provided	20/138 (14%)	Plasma	Preoperative
ESCC	61	1	<65: n = 43; >65: n = 18	NA	TNM I–IV, Early (I/II): n = 15; Late (III/IV): n = 46	22/61 (36%).	Tissues	Postoperative

CRC, colorectal cancer; MC, multiple cancers; HNSCC, head and neck squamous cell carcinoma; BTC, biliary tract carcinoma; PC, prostate cancer; GC, gastric cancer; ESCC, esophageal squamous cell carcinoma; NA, not available.

a There were 562 CRC patients with TNM I–IV stage from included studies, but only 546 of them provided survival data.

### Association Between *mSEPT9* and OS in Cancer Patients

**Figure 2:**
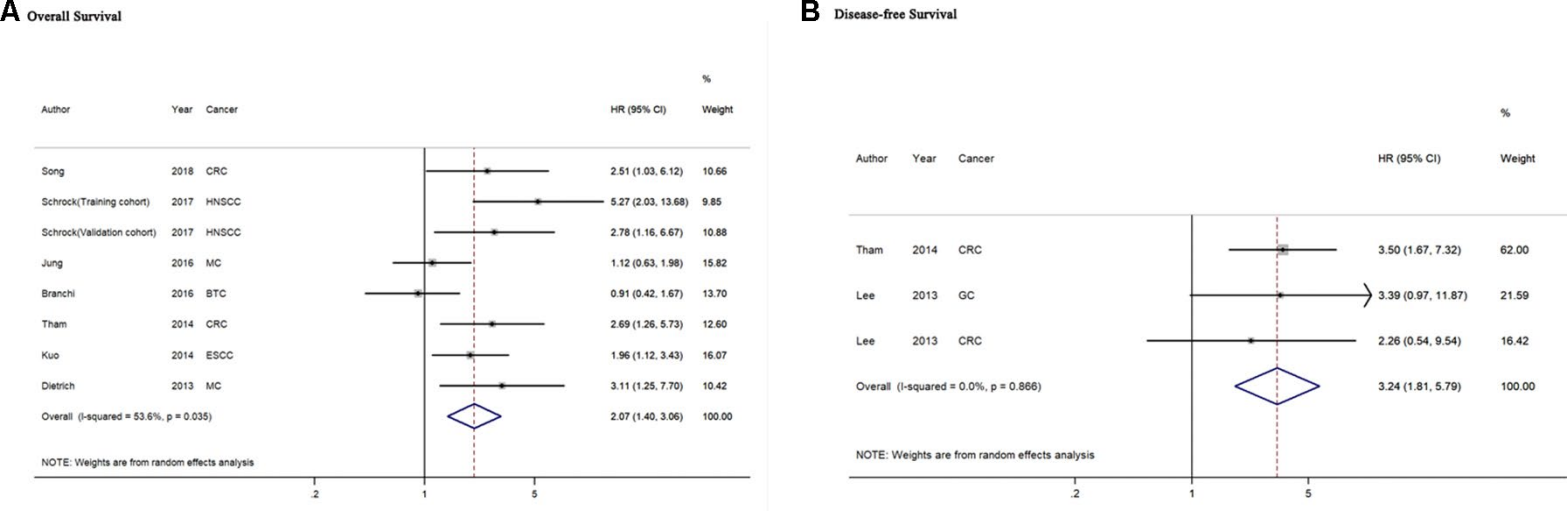
Forest plots for evaluation of the association between *mSEPT9* and overall survival **(A)** or disease-free survival **(B)** in cancer patients. BTC, biliary tract carcinoma; CRC, colorectal cancer; ESCC, esophageal squamous cell carcinoma; GC, gastric cancer; HNSCC, head and neck squamous cell carcinoma; MC, multiple cancers.

A total of seven studies including 786 cancer patients evaluated the association between *mSEPT9* and OS (Dietrich et al., 2013; Kuo et al., 2014; Tham et al., 2014; Branchi et al., 2016; Jung et al., 2016; Schrock et al., 2017; Song et al., 2018). The heterogeneity test showed high heterogeneity among these studies (*P_heterogeneity_* = 0.035, *I^2^* = 53.6%). The pooled HR estimated by a random-effects model was 2.07 (95% CI = 1.40–3.06), suggesting that *mSEPT9* was significantly associated with poor OS of cancer (**Figure 2A**). We further explored the prognostic role of *mSEPT9* in specific subgroups (**Table 3**). Results revealed that patients with *mSEPT9* detected in plasma or serum suffered reduced OS than those without (HR = 3.06, 95% CI = 1.99–4.70, *P_heterogeneity_* = 0.661, *I^2^* = 0%). Particularly, *mSEPT9* detected in preoperative plasma indicated a 3.25-fold increased risk of worse survival (95% CI = 1.93–5.48, *P_heterogeneity_* = 0.489, *I^2^* = 0%). We also performed a pooled analysis to summarize data from two studies of nonmetastatic CRC (I–III) and found decreased OS in *mSEPT9*-positive patients (HR = 2.61, 95% CI = 1.47–4.65).

**Table 3:** Subgroup analyses of the effects of mSEPT9 on overall survival of cancer patients.

Subgroup	Number of dataset	HR (95% CI)	P_heterogeneity_	*I^2^* (%)
Plasma/Serum	4	3.06 (1.99–4.70)	0.661	0
Preoperative^a^	3	3.25 (1.93–5.48)	0.489	0
CRC (I–III)^b^	2	2.61 (1.47–4.65)	–	–

CRC, colorectal cancer; HR, hazard ratios; CI, confidence interval.

a The sample type of the three included studies was preoperative plasma.

b Because there were only two included studies focusing on overall survival of CRC, pooled HR and 95% CI were estimated, and heterogeneity was not evaluated.

### Association Between *mSEPT9* and DFS in Cancer Patients

Two included studies comprising three datasets of 371 cancer patients reported the association of *mSEPT9*with DFS of cancer(Lee et al., 2013; Tham et al., 2014). The heterogeneity test showed no heterogeneity among these studies (*P_heterogeneity_* = 0.866, *I^2^* = 0%). The pooled HR of the aforementioned studies was 3.24 (95% CI = 1.81–5.79), indicating that *mSEPT9* predicted for worse DFS in cancer patients (**Figure 2B**). Subgroup analysis failed to be performed because of the limited number of relevant studies.

### Association Between *mSEPT9* and DSS/PFS in Cancer Patients

Only two studies reported the association of *mSEPT9* with DSS in cancer patients (Angulo et al., 2016; Freitas et al., 2018). Angulo et al. identified that *SEPT9* was hypermethylated in PC patients with a decreased DSS (HR = 7.64, 95% CI = 2.35–24.82) (Angulo et al., 2016). However, Freitas et al. reported that *mSEPT9* independently indicated an increased DSS in CRC patients (HR = 0.67, 95% CI = 0.47–0.97), and specially, *mSEPT9* was associated with a better DSS in colon cancer (HR = 0.47, 95% CI = 0.28–0.81) (Freitas et al., 2018).

For the *mSEPT9*’s prognostic role in PFS, Angulo et al. focusing on PC (HR = 2.52, 95% CI = 1.17–5.39) and Schrock et al. focusing on HNSCC (HR = 1.19, 95% CI = 1.10–1.56) both showed a significant association between *mSEPT9* and poor PFS of patients (Angulo et al., 2016; Schrock et al., 2017).

### Sensitivity Analyses and Publication Bias

Sensitivity analyses suggested that our pooled results were quite stable for both OS (**Supplementary Figure S1A**) and DFS (**Supplementary Figure S1B**). We observed a borderline significant publication bias in meta-analysis for OS (*P_Egger’s test_* = 0.048, *P_Begg’s test_* = 0.063). Therefore, we conducted a trim-and-fill analysis and found that despite publication bias, the adjusted pooled HR consistently demonstrated a significant association between *mSEPT9* and OS (HR = 1.61, 95% CI = 1.09–2.38, **Supplementary Figure S2**). There was no obvious publication bias for meta-analysis for DFS (*P_Egger’s test_* = 0.443, *P_Begg’s test_* = 0.296).

## Discussion

Several studies have investigated the association between *mSEPT9* and prognosis in human cancers, but results are uncertain due to the limited sample size and various cancer types. Herein, we conducted a systematic review and meta-analysis and supported that *mSEPT9* significantly predicted for worse cancer prognosis.

By systematic literature search, rigorous screening, and analysis, we identified that *mSEPT9*-positive cancer patients would suffer two-fold risk of decreased OS. Further subgroup analysis supported this result. Sensitivity analysis and trim-and-fill analysis guaranteed the robustness of our results. Specially, *mSEPT9* detected in preoperative plasma significantly indicated a worse OS, implying a convenient and promising way to predict long-term survival of cancer patients. In addition, our meta-analysis also supported that *mSEPT9* was significantly associated with poor DFS of cancer. Sensitivity analysis suggested that the result was stable, and Cochran’s Q test and *I^2^* statistic did not indicate considerable heterogeneity. The aforementioned results all suggested that *mSEPT9* could be a good prognostic biomarker for cancer patients. Traditionally, serum tumor markers (i.e., CEA, CA19-9) are used for screening and prognosis prediction, but their performance is still unsatisfactory. Previous studies have confirmed the excellent property of *mSEPT9* in early diagnosis of several cancers and have clearly elucidated the potential mechanisms (Church et al., 2014; Koch et al., 2018; Pan et al., 2018). Now we provide evidence to support that *mSEPT9* also could be a promising biomarker for cancer prognosis, which can be combined with traditional tumor biomarkers to greatly improve prognosis prediction in the future.

There were several limitations in our work. First, our results strongly supported that *mSEPT9* could be a prognostic indicator of OS and DFS for human cancer, but there were not enough studies for subgroup analysis to fully clarify its impact on different cancer types, sampling times, and pathological stages. Second, there were only two included studies about DSS and PFS. The limited number of studies impeded us to conduct a meta-analysis to evaluate the impact of *mSEPT9* on DSS and PFS. Last, some included studies did not provide multivariate-adjusted HRs, so we used unadjusted HRs instead. These unadjusted HRs were possibly influenced by potential confounders in the original studies. When we pooled them into a meta-analysis, the influence might be magnified and lead to a risk of bias on the pooled results. More studies with elaborate design should be conducted to verity our results and further explore more detailed impacts of *mSEPT9* on cancer prognosis.

## Conclusion

Our meta-analysis suggests that *mSEPT9* could predict for worse OS and DFS in cancer patients. Specially, patients with detection of *mSEPT9* in preoperative plasma would suffer significantly decreased OS of cancer. To the best of our knowledge, this is the first meta-analysis providing robust evidence that *mSEPT9* could be a promising biomarker for cancer prognosis.

## Data Availability

All datasets analyzed for this study are included in the manuscript and the **Supplementary Files**.

## Conflict of Interest Statement

The authors declare that the research was conducted in the absence of any commercial or financial relationships that could be construed as a potential conflict of interest.

## Author Contributions

HX and YL designed the study and revised the manuscript. NS designed the study, summarized the data, and wrote the manuscript. TW, DL, and YZ performed literature search, collected data, and performed some analysis. All authors read and approved the final manuscript.

## Supplementary Material

Supplementary Figure S1 Sensitivity analysis for overall survival **(A)** and disease-free survival **(B)**.

Supplementary Figure S2 Begg's funnel plots of overall survival before **(A)** and after **(B)** performing trim-and-fill analysis.

Supplementary Table S1 PRISMA 2009 Checklist.

Supplementary Table S2 Quality evaluation of included studies by Newcastle-Ottawa Scale.
