# Supplementary figures and images for: Hypermethylation of the SEPT9 Gene Suggests Significantly Poor Prognosis in Cancer Patients: A Systematic Review and Meta-Analysis
Source: Front Genet. 2019 Sep 19;10:887. doi: 10.3389/fgene.2019.00887 (PMC6761278; doi:10.3389/fgene.2019.00887)

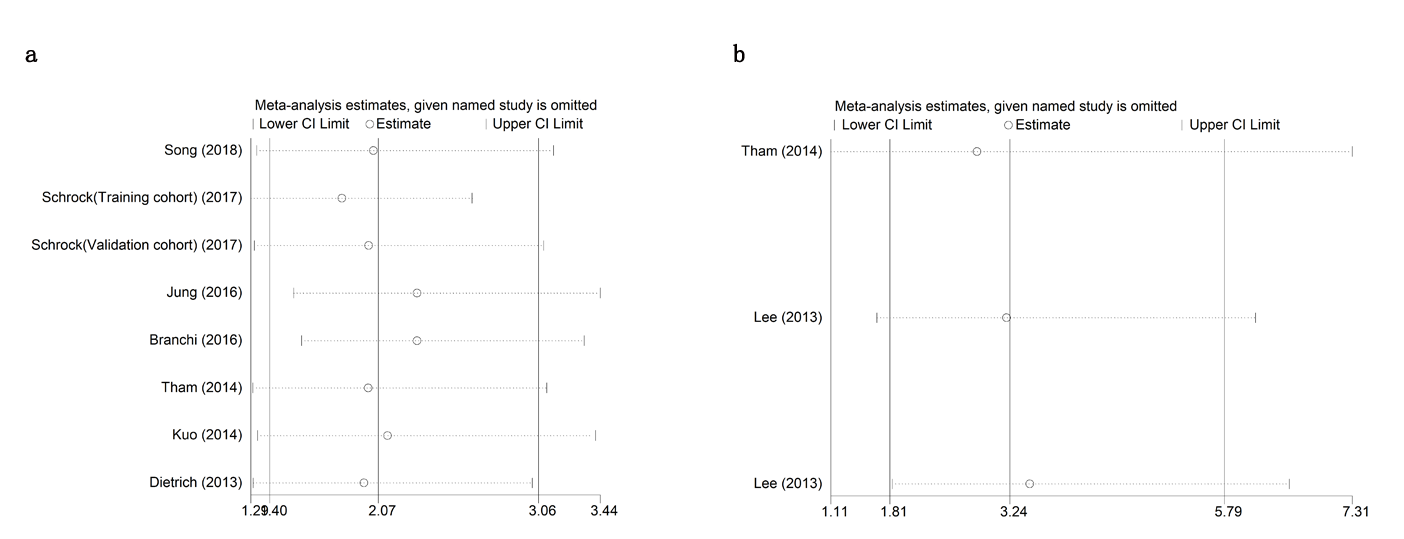

Supplement: Supplementary Figure S1 — Sensitivity analysis for overall survival (A) and disease-free survival (B). [file Image_1.tif]

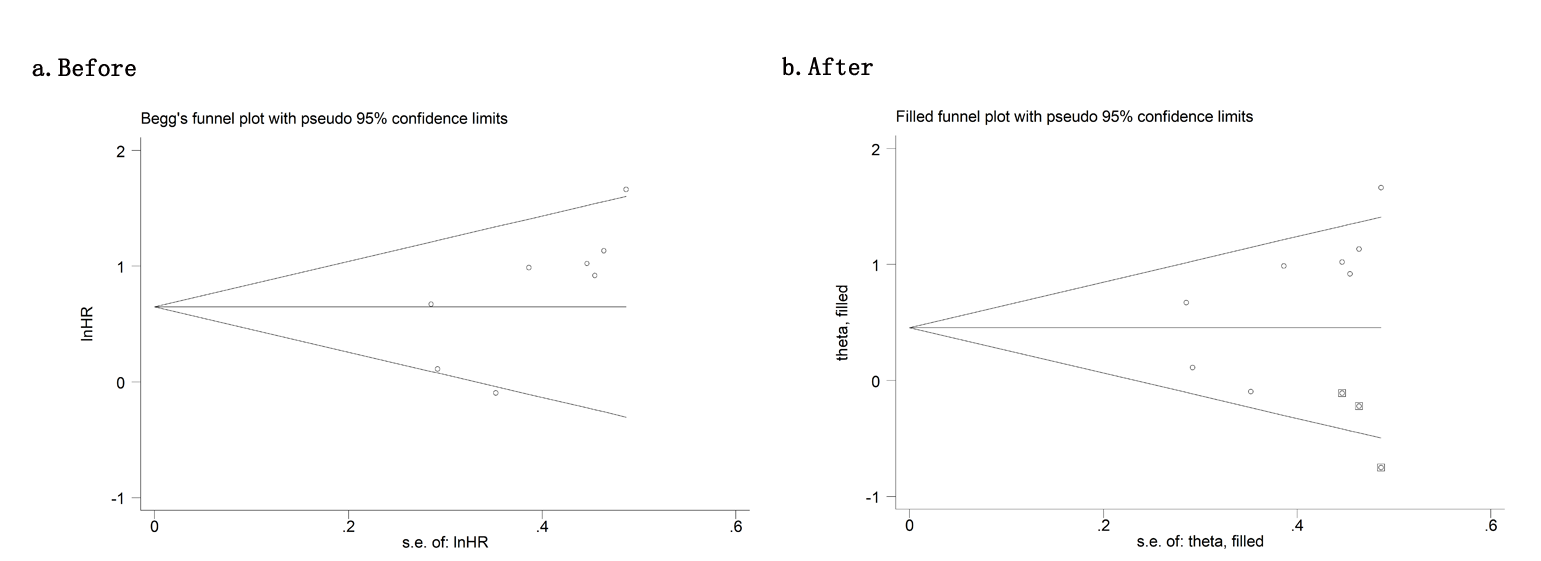

Supplement: Supplementary Figure S2 — Begg's funnel plots of overall survival before (A) and after (B) performing trim-and-fill analysis. [file Image_2.tif]
