# Supplementary material for: Hypermethylation of the SEPT9 Gene Suggests Significantly Poor Prognosis in Cancer Patients: A Systematic Review and Meta-Analysis
Source: Front Genet. 2019 Sep 19;10:887. doi: 10.3389/fgene.2019.00887 (PMC6761278; doi:10.3389/fgene.2019.00887)
Supplement: Supplementary Table S2 — Quality evaluation of included studies by Newcastle-Ottawa Scale. [file Table_2.doc]

Supplementary Table S2. Quality evaluation of included studies by Newcastle-Ottawa Scale

| Study | Selection (0-4) | | | | Comparability (0-2) | | Outcome (0-3) | | | Score |
| --- | --- | --- | --- | --- | --- | --- | --- | --- | --- | --- |
|  | REC | SNEC | AE | DO | SC | AF | AO | FU | AFU |  |
| Song (2018) | 0 | 1 | 1 | 1 | 0 | 0 | 1 | 0 | 0 | 4 |
| Freitas (2018) | 0 | 1 | 1 | 1 | 1 | 1 | 1 | 1 | 0 | 7 |
| Schrock (Training cohort) (2017) | 0 | 1 | 1 | 1 | 0 | 0 | 1 | 1 | 1 | 6 |
| Schrock (Validation cohort) (2017) | 0 | 1 | 1 | 1 | 0 | 0 | 1 | 1 | 1 | 6 |
| Jung (2016) | 0 | 1 | 1 | 1 | 0 | 0 | 1 | 1 | 0 | 5 |
| Branchi (2016) | 0 | 1 | 1 | 1 | 0 | 0 | 1 | 1 | 0 | 5 |
| Angulo (2016) | 0 | 1 | 1 | 1 | 1 | 1 | 1 | 1 | 1 | 8 |
| Tham (2014) | 0 | 1 | 1 | 1 | 1 | 1 | 1 | 1 | 1 | 8 |
| Kuo (2014) | 0 | 1 | 1 | 1 | 0 | 0 | 1 | 0 | 1 | 5 |
| Lee (2013)-GC | 0 | 1 | 1 | 1 | 0 | 0 | 1 | 0 | 1 | 5 |
| Lee (2013)-CRC | 0 | 1 | 1 | 1 | 0 | 0 | 1 | 0 | 1 | 5 |
| Dietrich (2013) | 0 | 1 | 1 | 1 | 0 | 0 | 1 | 0 | 1 | 5 |

Abbreviations: REC, representativeness of the exposed cohort; SNEC, selection of the non-exposed cohort; AE, ascertainment of exposure; DO, demonstration that outcome of interest was not present at start of study; SC, study controls for sex and age; AF, study controls for any additional factor (e.g., stage, histology); AO, assessment of outcome; FU, follow-up long enough for outcomes to occur; AFU, adequacy of follow up of cohorts.

Notes: “1” means that the study satisfied the item, and “0” means the opposite situation.
